# Variances in physiological parameters associated with stress tolerance between seven *Brassica oleracea* varieties

**DOI:** 10.3389/fpls.2025.1713155

**Published:** 2025-12-19

**Authors:** Dino Davosir, Ivana Šola, Jutta Ludwig-Müller

**Affiliations:** 1 Faculty of Biology, Technische Universität Dresden, Dresden, Germany; 2 Department of Biology, Faculty of Science, University of Zagreb, Zagreb, Croatia

**Keywords:** abiotic stress markers, antioxidants, Brassicaceae, flavonoids, glucosinolates, oxidative stress, polyphenols, stress tolerance

## Abstract

Economically important *Brassica oleracea* plants are increasingly used as an alternative to the traditionally used *Arabidopsis thaliana* as models in plant stress biology. However, the extensive diversity of *B. oleracea* varieties, belonging to different vegetable forms, is often overlooked. Due to previous results indicating that basal levels of stress parameters (reference baseline values in unstressed plants) are important predictors of stress tolerance, we selected seven varieties to comparatively analyze the basal levels of a wide array of stress parameters, intending to guide future studies. A high variability was observed between the varieties for most parameters, including osmolytes, photosynthetic pigments, and antioxidative parameters. Particular interest was given to specialized metabolites, such as phenolics and glucosinolates, with established links between metabolites and the corresponding biosynthesis gene expression levels. Among all varieties tested, cauliflower exhibited the highest levels of phenolic and other antioxidant parameters, suggesting it may be the most resistant to oxidative stress. Meanwhile, kohlrabi, Brussels sprout, and Savoy cabbage excelled in photosynthetic and glucosinolate-related parameters, indicating higher tolerance to stresses affecting photosynthesis and glucosinolate-driven stress responses. Our results set the ground for future stress application studies to deal with the observed *B. oleracea* variability accordingly. We concluded that no single parameter alone can be used as a reliable indicator of stress tolerance. Therefore, we recommend that future studies employ a broad range of parameters and varieties to evaluate responses to specific stresses with *B. oleracea* varieties as promising alternative plant models.

## 1 Introduction

Due to climate change and increasingly common weather extremes, agricultural food production is faced with novel challenges (Shahzad et al., 2021). Plants are increasingly exposed to various abiotic and biotic stresses, or more often, their combinations, which has skyrocketed new research topics in plant biology. The most studied types of abiotic stress are heat, cold, flooding, drought, and increased salinity (Zhang et al., 2022b; Šola et al., 2025b). Herein, various study designs and plant species are utilized. Most often used in these types of studies is the model plant *Arabidopsis thaliana*, due to its small size, short life cycle, high seed production, published genome sequence, and a large set of available mutant lines (Raza et al., 2020a). However, due to its lack of economic significance, other Brassicaceae plants are increasingly used as model plants instead (Yaschenko et al., 2024; Zhou et al., 2024) to study, for example, heat stress, cold stress, salinity stress, flooding, drought and various other stresses, as reviewed previously (Del Carmen Martínez-Ballesta et al., 2013; Kayum et al., 2016; Ahmar et al., 2019).

Generally, the Brassicaceae family consists of at least 372 genera and 4060 recognized species (Raza et al., 2020b). However, the family is primarily known for Cruciferous vegetables. These include species such as *Brassica oleracea* (cultivated as broccoli, cabbages, cauliflower, collard greens, and kale), *B. rapa* (Chinese cabbage, turnip, bok choy, etc.), *B. napus* (rapeseed), *Raphanus sativus* (common radish), and *Armoracia rusticana* (horseradish) (Šamec and Salopek-Sondi, 2018). Of all Brassicaceae family members, *B. oleracea* species are the most extensively bred (Greer et al., 2023). According to most interpretations, *B. oleracea* species consists of eight cultivar groups (mostly identified with varieties), but the most universally important, based on human consumption, are the varieties: broccoli (*B. oleracea* var. *italica*, cultivar group Botrytis), Brussels sprout (*B. oleracea* var. *gemmifera*, cultivar group Gemmifera), white cabbage (*B. oleracea* var. *capitata*, cultivar group Capitata), cauliflower (*B. oleracea* var. *botrytis*, cultivar group Botrytis), kale (*B. oleracea* var. *acephala*, cultivar group Acephala), kohlrabi (*B. oleracea* var. *gongylodes*, cultivar group Gongylodes) and Savoy cabbage (*B. oleracea* var. *sabauda*, cultivar group Capitata). Other edible varieties include gai lan (cultivar group Alboglabra), collard greens, Jersey cabbage, ornamental kale, Lacinato kale, marrow cabbage (cultivar group Acephala), Tronchuda kale (cultivar group Tronchuda), caulini (cultivar group Botrytis), and hybrids (kalette, broccoflower, broccolini). According to most interpretations, all the morphologically diverse varieties within the *B. oleracea* species originated from wild cabbage by domestication and breeding (Mabry et al., 2021). Recently, it was reported that diversification in *B. oleracea* is at least in part driven by large-scale gene expression alterations introduced by structural variation (Li et al., 2024). Comparative analyses utilizing different *Brassica* varieties recognized them as an attractive model for analyzing epigenetic changes a while ago (Braszewska-Zalewska et al., 2010). However, in plant abiotic stress research, a large variability between *B. oleracea* varieties is often ignored, since most studies use a representative from a single variety or genotype in their experimental setups.

Studies dealing with the response of plants to abiotic stress nowadays include modern transcriptomic, proteomic, metabolomic, and biochemical methods, as well as their combinations. However, to this day, the most utilized are still the traditional biochemical methods of assessing the impact of stress on plants by measuring various physiological parameters (Füzy et al., 2019; Galieni et al., 2021). These include determination of the level of lipid peroxidation through malondialdehyde content as an indicator of membrane damage due to oxidative stress, levels of osmolytes proline, glycine betaine, and sugars, activities of antioxidative enzymes, the content of non-enzymatic antioxidants, and estimations of photosynthetic pigment levels, etc (Venkidasamy et al., 2019). Still, combinations of these methods, coupled with newer methodologies, give valuable insights into plant responses to stress. In previous years, phenolic compounds were recognized for their role in stress response (Kumar et al., 2023), and have also been an important biomarker of stress conditions since. Additionally, in the family Brassicaceae, glucosinolates, another class of specialized metabolites, have been recognized to play an important role in abiotic and biotic stress tolerance (Variyar et al., 2014).

A previous study, utilizing a large selection of kale accessions, has examined the impact of individual osmotic stress, elevated temperature stress, and their combination. Their main conclusion was that accessions more tolerant to stress conditions were characterized by higher basal content (the reference values in unstressed, control plants) of proline, total sugars, glucosinolates, and higher transcription of *NAC* and *DREB* than less tolerant accessions (Bauer et al., 2022). Basal values of stress biomarkers appear to be an important predictor of stress tolerance in *Brassica*, making it essential to compare these values across varieties of interest to guide future studies and agricultural practices. Therefore, in this study, we selected seven varieties, commercially available representatives from each of the most important *B. oleracea* cultivar groups and compared the basal levels of often-used physiological parameters as biomarkers in stress research. The studied biomarkers were selected based on the relevant plant stress physiology literature and a bibliometric analysis of research papers investigating the stress responses of *B. oleracea* plants. We hypothesize that there will be a high variance in the tested parameters between varieties. Indications from our comparative analysis will be juxtaposed with the previous findings on the impact of abiotic stressors on different plant varieties studied, with the overall aim of highlighting the importance of utilizing a larger number of varieties in plant stress research.

## 2 Materials and methods

### 2.1 Bibliometric analysis

To investigate the research dynamics in Brassicaceae stress physiology, a preliminary bibliometric analysis was conducted. Tentative publication trends were ascertained using the Web of Science (WoS) Core Collection of the WoS database as the data source. The following Boolean topic search string was constructed (“species investigated” AND stress AND (heat OR freezing OR cold OR temperature OR drought OR flood OR waterlogging OR water OR osmotic OR salt OR UV OR oxidative OR pathogen OR biotic OR abiotic)), and total and publication counts for each year up to 2024 were taken directly for each species investigated. For the keyword co-occurrence analysis of research papers featuring *B. oleracea*, the results of the WoS query were manually filtered to exclude studies not relevant to plant stress research, as well as heavily applied and otherwise unrelated studies, based on the title and abstract, yielding a total of 330 studies (**Supplementary Table S1**). The dataset was imported into the VOSviewer tool (Van Eck and Waltman, 2010) to produce the keyword co-occurrence map, based on author keywords and WoS KeyWords Plus. The minimum number of keyword occurrences threshold was set to six.

### 2.2 Plant growth conditions

Seeds of broccoli (*B. oleracea* var. *italica*), Brussels sprout (*B. oleracea* var. *gemmifera*), cauliflower (*B. oleracea* var. *botrytis*), kale (*B. oleracea* var. *acephala*), kohlrabi (*B. oleracea* var. *gongylodes*), Savoy cabbage (*B. oleracea* var. *sabauda*), and white cabbage (*B. oleracea* var. *capitata*) were obtained from Agromlinar (Zagreb, Croatia), brand Vita Bella. Three semi-independent growth experiments were conducted, each starting one week apart, with growth periods overlapping. Seeds were sown in plastic pots with approximately 30 seeds per pot and five pots per variety on steam-sterilized soil substrate Stender B400 under greenhouse conditions (day temperatures 20-25°C, humidity ~65%). Plants were supplemented with artificial lighting to obtain a 16 h day/8 h night photoperiod and were regularly watered with tap water. After 7 days, seedlings were transplanted into individual nursery pots (Ø 9 cm, with holes at the bottom). On the 50^th^ day after sowing, overground parts of the plants were collected (pools of approximately 30 plants per biological replicate and variety) and immediately frozen in liquid nitrogen and stored at −80°C until freeze-drying, using an Alpha 1–2 lyophilizer (Martin Christ Gefriertrocknungsanlagen GmbH, Osterode am Harz, Germany) at −55 °C and 0.05 mbar for approximately 32 h. Lyophilized plant material was ground to a fine powder in a mortar with a pestle using liquid nitrogen. Sample aliquots were weighed and stored at −20°C in microcentrifuge tubes until downstream analyses.

### 2.3 Determination of metabolites and antioxidative capacity

Extraction was performed with 70% ethanol, using an equivalent of 30 mg dry weight (DW)/mL, as reported previously (Davosir and Šola, 2023). The plant material, mixed with the solvent, was shaken on a rotary shaker (SB3, Stuart Equipment, New Bern, NC, USA) at room temperature. After 60 min, the mixture was centrifuged for 5 min at 13000 g, and the supernatants were stored at −20°C for further analyses of the content of osmolytes, phenolics, glucosinolates, and antioxidative capacity. All downstream spectrophotometry measurements were performed on an Infinite^®^ 200 PRO microplate reader (Tecan, Männedorf, Switzerland). The detailed protocols are provided in the **Supplementary Material** file.

The content of proline and soluble sugars (SS) in the ethanolic extracts was determined using colorimetry-based assays by UV/Vis spectrophotometry, as described (Dubois et al., 1956; Bates et al., 1973), with modifications introduced in a previous study, the most important being that every sample had a separate blank to minimize the absorbance of other compounds present in the extract (Davosir et al., 2024b) (further details in **Supplementary Material 1.1, 1.2**). Solutions of known concentrations of L-proline and sucrose, respectively, were used for the indirect estimation of each metabolite in the extracts.

The content of specialized metabolites from ethanolic extracts was determined spectrophotometrically, using authentic standards for the indirect estimation of each compound class. Total phenolic (TP) content and content of phenolic classes (total flavonoids, TF; flavonols, TFl; phenolic acids, TPA; hydroxycinnamic acids, THCA; anthocyanins, TA) were determined using the previously described protocols (Singleton and Rossi, 1965; Zhishen et al., 1999; Howard et al., 2003; Lee et al., 2005; Jain et al., 2017; Šola et al., 2022; Davosir and Šola, 2023; Davosir et al., 2024a). As standards for each method, solutions of gallic acid, quercetin, caffeic acid, and pelargonidin chloride were used, respectively (**Supplementary Material 1.3-1.7**). The total glucosinolates (TGls) content was determined using a colorimetry-based method that utilizes sodium tetrachloropalladate, with sinigrin solutions as standards, according to a reported protocol (Mawlong et al., 2017), as described in detail (**Supplementary Material 1.8**).

Analysis of the antioxidative capacity (AC) was performed using the ABTS (2, 2′-azino-bis(3-ethylbenzothiazoline-6-sulfonic acid) radical scavenging assay, DPPH (2, 2-diphenyl-1-picrylhydrazyl) radical scavenging assay, and FRAP (ferric ion reducing antioxidant power) assay, as previously reported (Benzie and Strain, 1999; Re et al., 1999; Germanò et al., 2002), with some modifications (Šola et al., 2023), as described in **Supplementary Material 1.9-1.11**. Results were reported as L-ascorbic acid (AA) equivalents, calculated from the antioxidative capacity of the AA standard solutions.

### 2.4 Extraction and determination of photosynthetic pigment content

Photosynthetic pigments (chlorophylls and total carotenoids) were extracted from 15 mg of plant material using 80% acetone as a solvent, under dim light. Plant material was mixed with 1 mL of solvent for 20 sec, followed by centrifugation for 5 min at 13000 g and 4°C. The supernatant was transferred to a new tube on ice. The same procedure was repeated twice more, until the pellet was colorless, with the supernatants from each repetition pooled together. The photosynthetic pigment content was analyzed by measuring the absorbance of the extracts at specific wavelengths, and the content of pigments was calculated as described previously for chlorophyll *a*, chlorophyll *b* and total carotenoids (Lichtenthaler, 1987). Individual carotenoids (β-carotene and lycopene) were analyzed and calculated as described (Nour et al., 2015), and total porphyrin content was analyzed as reported (Sarropoulou et al., 2012).

### 2.5 RNA extraction and gene expression analysis

RNA was extracted from lyophilized plant material following a previously developed protocol (Kumar et al., 2007), adapted for smaller volumes (Davosir et al., 2025). RNA was quantified in the extracts using the Nanodrop ND-1000 (Thermo Fisher Scientific, Waltham, MA, USA), and RNA integrity was checked on agarose gel. Extracts were treated with DNase I (Thermo Fisher Scientific, Waltham, MA, USA) according to the protocol provided by the manufacturer, followed by cDNA synthesis performed with the Maxima First Strand cDNA Synthesis Kit for RT-qPCR (Thermo Fisher Scientific, Waltham, MA, USA), according to the manufacturer’s instructions. Prepared cDNA diluted 1:10 was used in qPCR reactions. qPCRs were performed with a CFX96™ Real-Time System (Bio-Rad, Hercules, CA, USA) and qTOWER 2.2. (Analytik Jena, Jena, Germany), using the 2x qPCRBIO SyGreen Mix (PCR Biosystems, London, UK) and the primers reported in **Supplementary Table S2**. The PCR conditions were as follows: 3 min at 95°C, 40 cycles of 10 sec at 95°C and 30 sec at 60°C, followed by generating a melting curve from 65 to 95°C. PCRs were run in triplicate for each biological replicate. The obtained cycle threshold (Ct) values of the genes of interest were normalized to the Ct values of two reference genes, yielding the ΔCt values. The values were transformed, and the final results were reported as 2^-ΔCt^ values.

### 2.6 Data analysis

The data was statistically analyzed using STATISTICA 12 (StatSoft Inc., USA). Growth and experiments were performed in three replicates (pools of approximately 30 plants per replicate were collected), i.e., the biological experimental unit was the pool of plants from individual growth experiments, not individual plants. For the metabolic parameters, data consisted of measurements from three extractions from each growth experiment (*n* = 9). For gene expression data, the mean value of three technical replicates for each growth experiment was used in statistical analysis (*n* = 3). A statistical comparison between the varieties studied was conducted using one-way analysis of variance (ANOVA), followed by a *post hoc* Tukey’s honestly significant difference test (Tukey’s test). The differences between samples at *p* ≤ 0.05 were considered statistically significant. Pearson’s correlation coefficients (*r*) were calculated, with statistical significance at *p* ≤ 0.05. Principal component analysis (PCA) was performed on the matrix of mean values of parameters for each biological replicate separately (**Supplementary Table S3b**), with the data standardized (z-scaled), and visualized by plotting the two factors or principal components, which explained the highest portions of the variance. Hierarchical clustering analysis (HCA) was performed on the mean values using the average linking method, based on Euclidean distance as a measurement of distance.

## 3 Results and discussion

**Figure 1:**
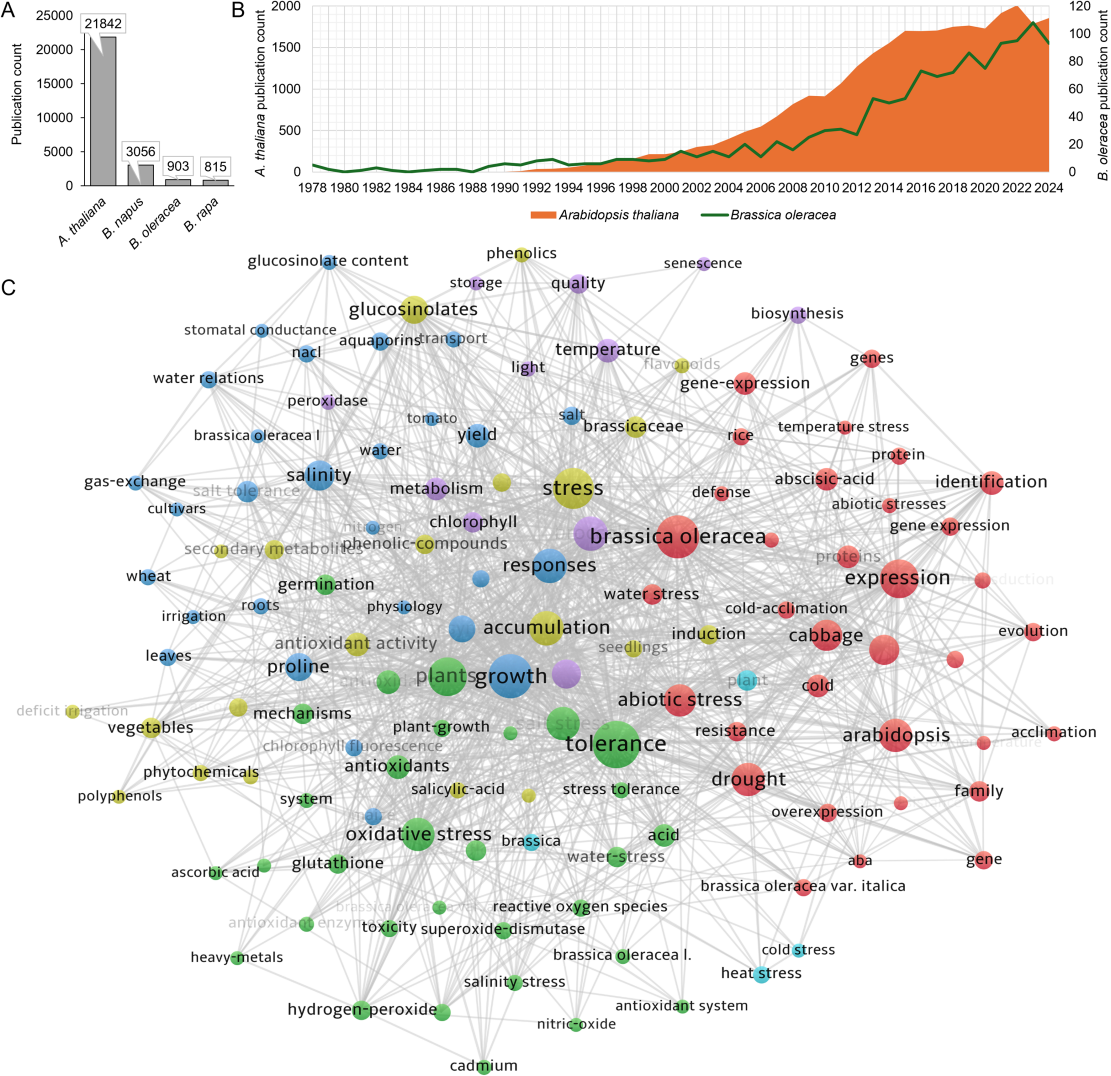
**(A)** Publication counts of papers featuring stress-related keywords and *Arabidopsis thaliana*, *Brassica napus*, *B. oleracea*, or *B. rapa* as research terms, derived from the Web of Science database search. **(B)** Yearly publication trends of research featuring stress-related keywords for *A. thaliana* and *B. oleracea*. **(C)** Keyword co-occurrence analysis of research study selection featuring *B. oleracea* stress-related research papers. The terms are displayed as derived from the data source, which may include variations in using capital letters, spelling, or formatting.

Our Web of Science (WoS) database search, utilizing pertinent keywords, revealed an increasing trend in stress physiology studies employing *B. oleracea* as models over the years (**Figure 1B**). Still, *A. thaliana* is by far a more popular model organism in plant stress research (**Figure 1A**). Also, among other Brassicaceae models of bigger economic significance, *B. napus* is more widely utilized compared to *B. oleracea* and *B. rapa*. Despite that, we propose that employing a selection of different *B. oleracea* varieties as models in research studies enables valuable insights into the variety-specific differences due to the rich diversity of this species, compared to representatives from the equally economically important but less diverse *B. napus*, a polyploid hybrid species between *B. oleracea* and *B. rapa*. Also, polyploidy can obscure gene expression patterns and complicate the interpretation of results, making *B. napus* a less ideal candidate for an alternative Brassicaceae model.

Traditional plant stress research suffers from a lack of high-throughput protocols, forcing authors to choose between a larger set of plant species or a broader range of experimental methods. The former problem is notable in studies with *B. oleracea* species as models, where a large variability of forms is present. In this study, we aimed to conduct the first comprehensive comparative analysis of the variety-specific differences in a broader range of physiological parameters commonly used in stress research. A considerable number of studies dealt with comparing various parameters between miscellaneous *B. oleracea* varieties, mostly from a nutritional perspective, as *Brassica* vegetables are important for human consumption. The novelty of our investigation lies in placing the comparative analysis of a large assortment of physiological parameters within the context of the potential stress tolerance of varieties with high basal levels of certain factors. The plant developmental stage also plays an important role in stress tolerance, with multiple studies confirming this (Rankenberg et al., 2021). However, given our focus on the variety-dependent differences, we directed our analysis to one developmental stage, i.e., younger plants, as early developmental stages are more vulnerable to stress conditions (Rankenberg et al., 2021).

A keyword co-occurrence analysis of studies dealing with *B. oleracea* stress responses assisted us in determining a selection of stress parameters to compare across varieties. Glucosinolates and proline emerged as the most occurring biomarker-related keywords (**Figure 1C**, **Supplementary Table S4**). The following were abscisic acid, antioxidative activity, chlorophyll, glutathione, hydrogen peroxide, phenolic compounds, lipid peroxidation, antioxidative enzymes, and ascorbic acid. Drawing on the keyword co-occurrence analysis, relevant literature, and methodological boundaries, we identified a set of stress parameters for assessment. We examined the levels of proline and soluble sugars, photosynthetic pigments, and the status of the antioxidative system. Furthermore, we investigated the accumulation of phenolic compounds and glucosinolates, specialized metabolites involved in stress response in *B. oleracea*. Additionally, metabolic data were coupled with complementary gene expression results to gain deeper insights into the transcriptional regulation of biosynthesis of the metabolites and processes studied in *B. oleracea* varieties. Furthermore, young *B. oleracea* plants (often branded as “baby greens”) are increasingly present in the human diet, with phenolics and glucosinolates analyzed in our study being noteworthy as health-promoting compounds (Björkman et al., 2011). Therefore, our results are also valuable from a nutritional perspective, as this comparative analysis will reveal which varieties are richer in specific compounds (Baenas et al., 2012).

### 3.1 Osmolytes

**Figure 2:**
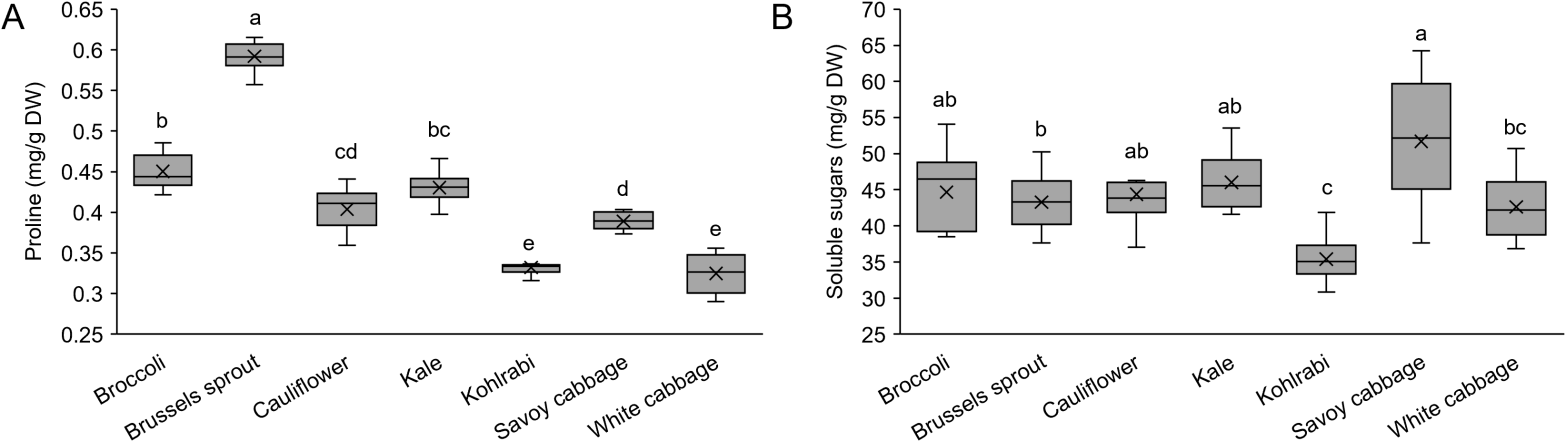
Content of **(A)** proline and **(B)** soluble sugars in seven *Brassica oleracea* varieties. Values represent the mean ± standard deviation (*n* = 9). Different letters indicate a significant difference between each variety (ANOVA, Tukey’s test, *p* ≤ 0.05). DW, dry weight.

Soluble sugars and, particularly, proline are often used as trusted biomarkers of stress conditions in plants (Suprasanna et al., 2015). They both act as compatible osmolytes during stress conditions and have been shown as an effective component of stress tolerance. Generally, they protect plants from stress through multiple routes by contributing to cellular osmotic adjustment, detoxifying reactive oxygen species (ROS), protecting membrane integrity, and stabilizing proteins (Ashraf and Foolad, 2007). Previous studies reported that heat stress (Cha et al., 2020) and hot-water treatment (Šola et al., 2023) increase the proline content in broccoli, but drought stress (Sardar et al., 2024) and cold-water treatment (Šola et al., 2023) have the opposite effect. Cold stress (Atici et al., 2003), heat stress (Hossain et al., 1995), salt and drought (Sahin et al., 2018) stimulate proline synthesis in cabbage. Furthermore, proline application has been reported to alleviate drought stress in cauliflower (El-Bauome et al., 2022) and salt stress in cabbage (Hiji and Jerry, 2020). Our results revealed variable proline levels across the selected varieties (**Figure 2a**). Variable proline levels were also observed between different kale accessions (Bauer et al., 2022), rice cultivars (Joseph et al., 2015), maize hybrids (Špoljarević et al., 2011), and barley cultivars (Naidu et al., 1992). Such variable examples for a given stress metabolite could therefore be expected, and these may be attributed to genetic differences. In *A. thaliana* accessions, genetic variations of the proline synthesis enzyme Δ^1^-pyrroline-5-carboxylate synthetase 1 were linked with different proline accumulation levels (Kesari et al., 2012). In our study, Brussels sprout had the highest proline content, while kohlrabi and white cabbage had the lowest. A previous study comparing kale and white cabbage also recorded lower basal proline levels in white cabbage (Pavlović et al., 2019). However, when challenged with salinity stress, kale accumulated less proline than the less tolerant white cabbage. Nevertheless, since higher proline levels are generally associated with higher stress tolerance, further research should confirm whether Brussels sprout could be less susceptible to various stresses, particularly salt stress (El-Bauome et al., 2022), due to its high basal proline content.

As shown in **Supplementary Table S5**, SS levels displayed less variability between varieties (*F* = 7.69) when compared to proline (*F* = 192.07). In fact, based on the *F*-values and *p*-values of the ANOVA analysis, SS levels displayed the lowest variability between varieties out of all the evaluated parameters. This could be due to the central and conserved role of SS in the primary metabolism and multiple cellular processes, compared to other parameters tested. In tomatoes, a set of quantitative trait loci (QTLs) was identified to explain the differences in SS accumulation between varieties (Tang et al., 2021). Since our selection of *B. oleracea* varieties displayed a low variance in SS levels, it could indicate a relatively smaller genetic variability of SS-related QTLs. Despite low variability between the varieties, SS levels are highly susceptible to environmental stresses. This was demonstrated in cauliflower (Collado-González et al., 2021) and kale (Bauer et al., 2022) during heat stress, kale during cold stress (Steindal et al., 2015), and broccoli during salinity stress, high and low growing temperatures (Zaghdoud et al., 2012; Gmižić et al., 2023; Šola et al., 2024a). Similarly to proline, the lowest SS values were recorded in kohlrabi and white cabbage (**Figure 2b**). The link between the metabolism of proline and SS was previously suggested (Gurrieri et al., 2020), and could be the reason for the similar trends between proline and SS. Similar to our results, a study comparing the phytochemical composition of various Brassicaceae seedlings also recorded the lowest SS levels in kohlrabi when compared to Brussels sprout, cauliflower, and Savoy cabbage (Šola et al., 2024b). This suggests that similar SS level patterns are maintained throughout earlier developmental stages, i.e., from seedlings to younger vegetative plants. Some studies previously associated high SS levels with higher stress tolerance (Sami et al., 2016; Bauer et al., 2022). Lower levels of both SS and proline in kohlrabi and white cabbage may indicate their lower stress tolerance. However, despite the connection between stress tolerance and osmolyte accumulation, a causal relationship between the two is not generally affirmed (Ahmad et al., 2020).

### 3.2 Photosynthetic pigments

**Figure 3:**
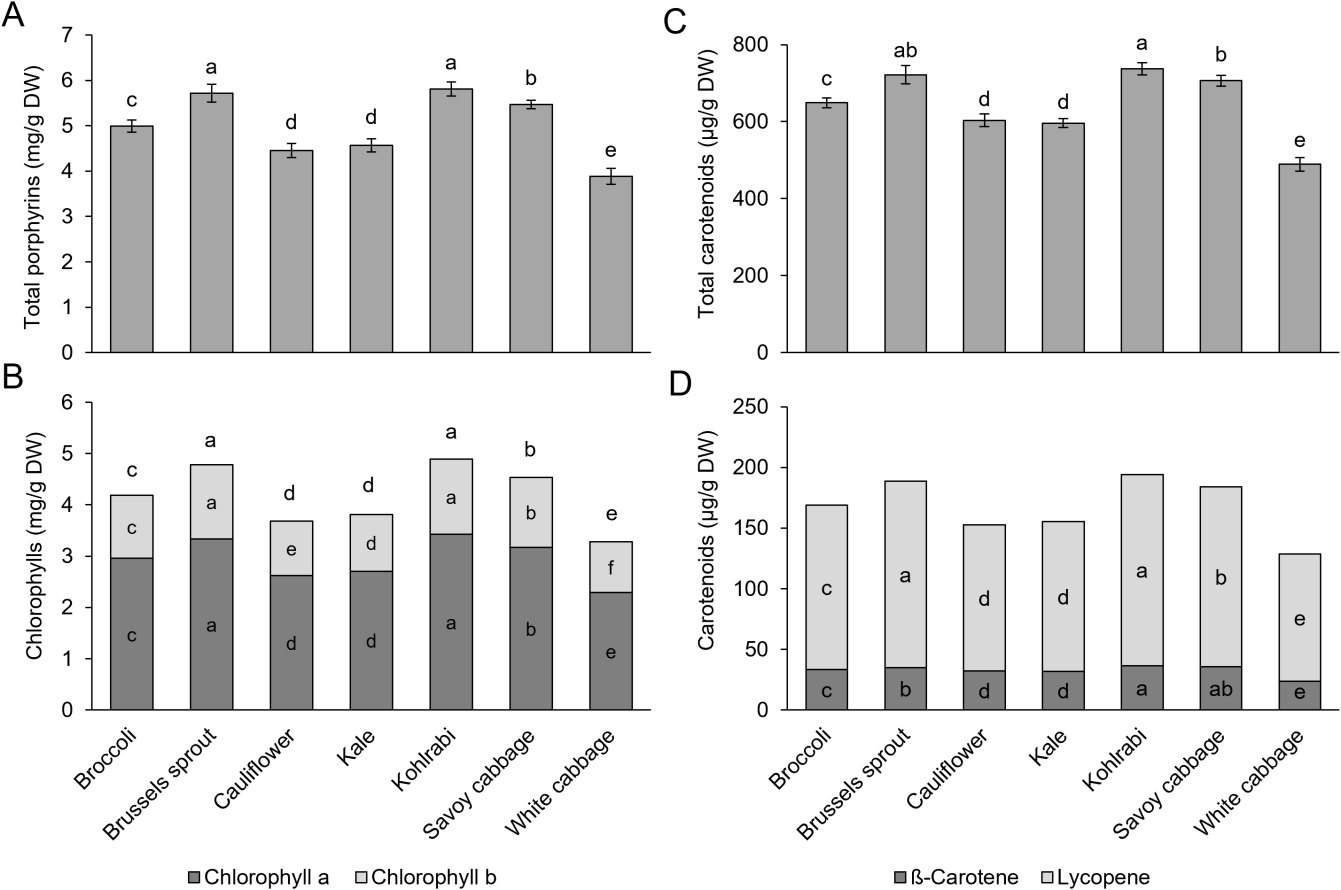
Pigment content in seven *Brassica oleracea* varieties: **(A)** total porphyrins, **(B)** chlorophyll *a* and *b*, and total chlorophyll (chlorophyll *a* + *b*), **(C)** total carotenoids, **(D)** individual carotenoids, ß-carotene, and lycopene. Values represent the mean **(B-D)** ± standard deviation **(A-C)** (*n* = 9). Different letters indicate a significant difference between each variety (ANOVA, Tukey’s test, *p* ≤ 0.05). DW, dry weight. Exact values are reported in the **Supplementary Table S6**.

Photosynthesis is one of the main biochemical processes in most plant cells. Changes in the photosynthesis rate are often linked to stress conditions in plants. Photosynthetic pigments are particularly prone to oxidative stress in plant cells, and their degradation is frequently monitored when assessing the stress conditions in plants (Chauhan et al., 2023). Multiple studies reported the negative influence of stress conditions on photosynthetic pigments in *B. oleracea*, as demonstrated for temperature stresses (Soengas et al., 2018; Šola et al., 2023), heavy-metal stress (Natasha et al., 2022), waterlogging (Brazel et al., 2024), and drought (Sardar et al., 2024). As previously reported, the content of photosynthetic pigments can vary based on the *Brassica* cultivar studied (Kopsell et al., 2004; Korus and Kmiecik, 2007). Furthermore, pigment level and composition change drastically through leaf ontogeny, as demonstrated for kale (Lefsrud et al., 2007) and broccoli (Gmižić and Šola, 2025). We also observed variable levels of photosynthetic pigments between different varieties (**Figure 3**; **Supplementary Table S6**). Similarly, as for the previous parameters studied, higher photosynthetic pigment levels were linked to higher stress tolerance in various species (Majid Khayatnezhad, 2012; Syamsia et al., 2018; Ehumadu et al., 2024). Brussels sprout and kohlrabi had the highest levels of most photosynthetic pigment parameters, while in white cabbage plants, the lowest levels were observed. When compared with broccoli and kale, white cabbage sprouts had the lowest levels of photosynthetic pigments (Šamec et al., 2018), corroborating our results. Furthermore, broccoli microgreens also had higher levels of chlorophylls, carotenoids, and porphyrins, the latter values include also precursors and intermediates of chlorophyll biosynthesis and other minor porphyrins, than cauliflower and kale (Davosir and Šola, 2023). Most photosynthetic pigment parameters positively correlated with each other (**Supplementary Table S7**). This was previously observed in multiple studies (Farnham and Kopsell, 2009; Zhang et al., 2022a; Izadpanah et al., 2024), and can probably be linked with functional interconnection and the shared early biosynthesis pathways of chlorophylls and carotenoids, which diverge after the formation of geranylgeranyl pyrophosphate (Liu et al., 2020). Interestingly, although kohlrabi displayed the lowest levels of proline and SS, it also had the highest photosynthetic pigment levels. In general, it is known that lower sugar levels enhance photosynthesis (Rosa et al., 2009). In this case, it could be through the increase in the synthesis of photosynthetic pigments. Also, high photosynthetic pigment content caused a high level of SS. Under those conditions, plants promote the export of SS to sink tissues and starch synthesis (Rosa et al., 2009), which could be the reason for the lower SS content in kohlrabi. During stress conditions, high photosynthetic pigment levels in kohlrabi plants may compensate for the low osmolyte levels. This also confirms that no single parameter should be used to assess the tolerance of different varieties and points to the high metabolic plasticity and variability of *B. oleracea* varieties.

### 3.3 Antioxidative system

**Figure 4:**
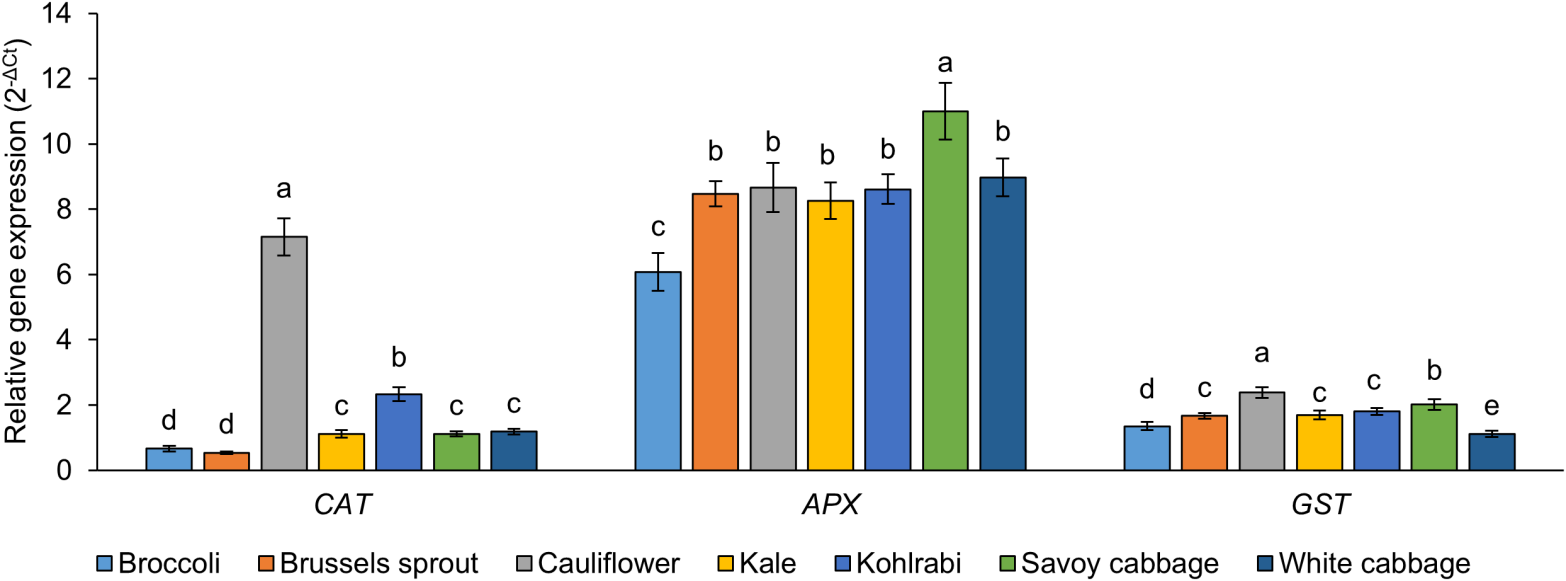
Expression of genes coding for enzymes of the antioxidative system, catalase (CAT), ascorbate peroxidase (APX), and glutathione *S*-transferase (GST) in seven *Brassica oleracea* varieties. Values are reported as 2^-ΔCt^, normalised to the expression of two reference genes. Values represent the mean ± standard deviation (*n* = 3). Different letters indicate a significant difference between each variety (ANOVA, Tukey’s test, *p* ≤ 0.05).

Upregulation of the plant antioxidative system is a common occurrence during stress conditions in plants and is therefore a valuable biomarker of stress (Noctor et al., 2016). Often, various stress conditions cause an imbalance of ROS in plant cells. To combat the increase in ROS, the antioxidative system is often upregulated. One branch of the antioxidative system are the enzymatic antioxidants (catalases, peroxidases, superoxide dismutases, glutathione *S*-transferases, glutathione reductases) (Mishra et al., 2023). To study the antioxidative system of *B. oleracea* varieties, we measured the expression of genes encoding isoforms of catalase (CAT), ascorbate peroxidase (APX), and glutathione *S*-transferase (GST) (**Figure 4**). These genes were selected because they were investigated in previous studies related to stress responses in *B. oleracea* (Bernard et al., 2016; Zheng et al., 2019). The highest *CAT* expression was recorded in cauliflower, while significantly lower expression values were observed in all other varieties, with the lowest levels in broccoli and Brussels sprout. A previous study compared the enzymatic activity of CAT in kale and white cabbage, and yielded the same results as recorded in the current study for *CAT* gene expression (Soengas et al., 2018). Broccoli also had the lowest *APX* gene expression, with the highest recorded in Savoy cabbage. Similar basal CAT and APX activity patterns were observed in Savoy cabbage and white cabbage. However, when challenged with increased salinity, Savoy cabbage answered with a higher increase of CAT and APX activity (Sanoubar et al., 2016). Furthermore, higher relative *APX* gene expression compared to *CAT* aligns with a prior report of APX exhibiting higher activity than CAT in *B. oleracea* (Zaimoglu et al., 2011). For *GST*, the highest expression was recorded in cauliflower, and the lowest in white cabbage. Since most abiotic and biotic stressors in plants result in oxidative stress conditions (Demidchik, 2015), the observation that cauliflower has the highest expression for two of the three genes analyzed may indicate that the variety has a more robust enzymatic antioxidant machinery, making it more resilient to oxidative stress.

**Figure 5:**
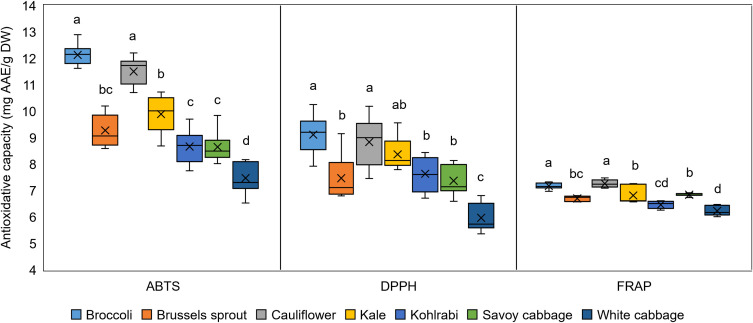
Antioxidative capacity of extracts from seven *Brassica oleracea* varieties, measured by ABTS, DPPH, and FRAP assays. Values represent the mean ± standard deviation (*n* = 9). Different letters indicate a significant difference between each variety (ANOVA, Tukey’s test, *p* ≤ 0.05). AAE, *L*-ascorbic acid equivalents; DW, dry weight.

The other branch of the plant antioxidative system involves non-enzymatic antioxidants, including ascorbate, glutathione, tocopherol, carotenoids, phenolic compounds (polyphenols), and proline (Mishra et al., 2023). The total content of non-enzymatic antioxidants was measured in extracts of *B. oleracea* varieties by ABTS, DPPH, and FRAP antioxidative capacity (AC) assays (**Figure 5**). The results obtained by all three assays were mutually correlated (**Supplementary Table S7**), therefore validating the results. Furthermore, those results showed a generally lower distinction between varieties compared to the analyses of the enzymatic branch. Measurements of AC encompass a wide range of compounds (Huang et al., 2005), so a larger variability is expected than for the more specific measurements of a particular enzyme’s gene expression. In all the assays employed, broccoli and cauliflower had the highest AC values, while the lowest was recorded in white cabbage. High AC of broccoli was previously recorded (Jaiswal et al., 2011). A previous study (Soare et al., 2017) revealed that compared with five other varieties, white cabbage also had the lowest AC measured by the ABTS assay. The lowest AC of white cabbage among different varieties was also confirmed by further studies (Podsedek et al., 2006; Jaiswal et al., 2011; Voća et al., 2018). Similar to our results, when measured by ABTS, cauliflower sprouts also had the highest AC when compared with Brussels sprout, kohlrabi, and Savoy cabbage, which mutually had similar values (Šola et al., 2024b). High AC was also linked with the degree of stress tolerance in numerous studies (Dominguez-Perles et al., 2011; Weidner et al., 2011; Chutipaijit, 2016). Therefore, it could also be an important predictor of stress tolerance for broccoli and cauliflower plants and could point to lower stress tolerance of white cabbage. On one hand, the results also reveal that higher levels of non-enzymatic antioxidants in broccoli possibly compensate for the lower levels of enzymatic antioxidants, based on the observed gene expression patterns. On the other hand, cauliflower emerged with high levels of both branches of antioxidants compared to other varieties.

### 3.4 Phenolic compounds

**Figure 6:**
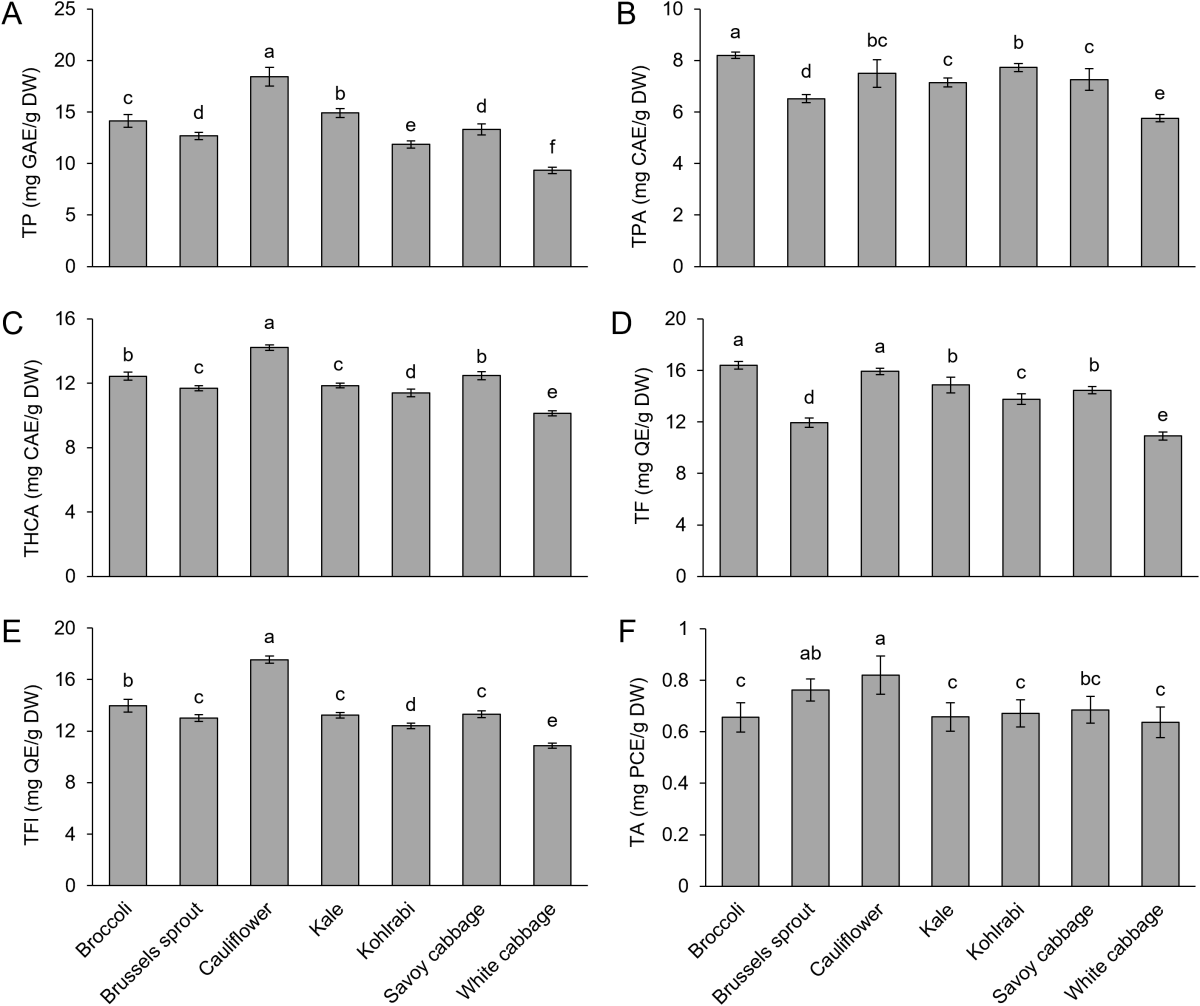
Content of **(A)** total phenolics (TP), **(B)** total phenolic acids (TPA), **(C)** total hydroxycinnamic acids (THCA), **(D)** total flavonoids (TF), **(E)** total flavonols (TFl), and **(F)** total anthocyanins (TA) in seven *Brassica oleracea* varieties. Values represent the mean ± standard deviation (*n* = 9). Different letters indicate a significant difference between each variety (ANOVA, Tukey’s test, *p* ≤ 0.05). GAE, gallic acid equivalents; DW, dry weight; CAE, caffeic acid equivalents; QE, quercetin equivalents; PCE, pelargonidin chloride equivalents.

Since phenolic compounds are among the main antioxidants in plants, we decided to analyze their content in more detail to evaluate their variability across different varieties. Phenolic compounds have emerged as important biomarkers of stress tolerance due to new insights into their role in managing abiotic and biotic stresses (Naikoo et al., 2019; Tuladhar et al., 2021; Šamec et al., 2021). Although the analysis of total phenolics (TP) in *B. oleracea* plants challenged with various stresses often did not yield any differences between controls and treated groups in multiple studies (Bauer et al., 2022; Huđ et al., 2023), we detected a significant variability in TP values across the tested varieties. Cauliflower emerged with the highest TP level, followed by kale, broccoli, Brussels sprout, Savoy cabbage, kohlrabi, and white cabbage with the lowest TP level (**Figure 6a**). When kale and white cabbage were subjected to cold stress, kale accumulated more TP, while upon heat stress, TP levels were the same between kale and white cabbage (Soengas et al., 2018). It was reported that the levels of phenolic subclasses and individual phenolic compounds in *Brassica* species reveal larger perturbations in phenolic biosynthesis during various stress conditions than the levels of TP (Muthusamy and Lee, 2024). Moreover, recently it was shown that high and low environmental temperatures induce specific shifts in the structural forms of phenolic compounds (Šola and Gmižić, 2025). Therefore, we also analyzed the content of major classes and subclasses of phenolic compounds (total flavonoids and their subclasses, flavonols and anthocyanins, and total phenolic acids with hydroxycinnamic acids as their subclass). A similar pattern to that for TP was observed for most phenolic (sub)classes (**Figure 6**), which can also be inferred from their mutual correlations (**Supplementary Table S7**). This points to the master regulation of the phenylpropanoid pathway, potentially early in the biosynthesis pathway. Levels of TPA between varieties did not align perfectly with the levels of THCA. These differences could originate from the different patterns of hydroxybenzoic acid (THBA) levels, which are another subclass of phenolic acids. It was also previously reported that two subclasses of phenolic acids differentially responded to various stresses. For example, under salinity stress, THCA levels increased and correlated with stress tolerance in kale and white cabbage, while THBA levels decreased (Muthusamy and Lee, 2024). Variable TF and TFl levels across varieties were observed, which is in accordance with the results of previous studies (Jahangir et al., 2009; Schmidt et al., 2010). However, our results in the present study slightly differ from the values of TF and TFl recorded in younger microgreens (Šola et al., 2024b). This indicates that the level of flavonoids varies across growth stages. Additionally, the TF patterns differ between species when initial/early plant growth stages are compared (Arena et al., 2024). Flavonoids are reported to be responsive to various stress conditions in *B. oleracea* (Muthusamy and Lee, 2024), and their application can sometimes alleviate stress-induced damage (Rodrigues De Queiroz et al., 2023). Similarly, high endogenous flavonoid levels, as observed in broccoli and cauliflower, could also have stress-attenuating properties during stress conditions. Since flavonoid biosynthesis is considered to be energetically expensive (Khan et al., 2012), the induction of the flavonoid pathway during stress conditions must be an important defense mechanism. Of all the subclasses, TA levels displayed the least variability between varieties (*F* = 12.64). Since anthocyanins are relatively scarce in the leaves of varieties studied compared to “red” or “purple varieties” (Park et al., 2017; Voća et al., 2018), it could be that most of them only maintain a similar, basal level of anthocyanins, which yielded the result of small variability between screened varieties. Furthermore, it is noteworthy to outline that broccoli and cauliflower, which fell at the higher end of the spectrum for most phenolic levels, are not only potentially more stress-tolerant but also stand out nutritionally as excellent sources of beneficial phenolics.

Due to their high antioxidative activity (Zeb, 2020), it is unsurprising that TP values and the values of most phenolic subclasses correlated with the results of AC measurements. Therefore, their role as essential antioxidants in *B. oleracea* is further corroborated, indicating that antioxidative capacity is a primary effector of phenolic-mediated stress defense. Interestingly, although cauliflower had the highest levels of TP and most phenolic subclasses, their levels in broccoli were lower, despite similar or even higher AC levels in broccoli. It could be that the high AC of broccoli was due to other compounds. Namely, broccoli was previously reported to have significantly higher levels of a strong antioxidant, L-ascorbic acid (vitamin C), than cauliflower (Singh et al., 2007). Therefore, we hypothesize that this could be the reason for its high AC content. In contrast, low levels of TP and phenolic subclasses likely also contributed to the lowest AC of white cabbage. Furthermore, correlations were established between the expression of *CAT* and *GST* genes and phenolic levels, as well as the expression of phenolic biosynthesis genes, indicating the synergistic regulation of both the enzymatic and non-enzymatic antioxidant branches. Such correlations have also been established previously (Rangani et al., 2019). Furthermore, recent findings reveal that GST proteins are related to the phenylpropanoid pathway in *B. oleracea* (Vijayakumar et al., 2016).

**Figure 7:**
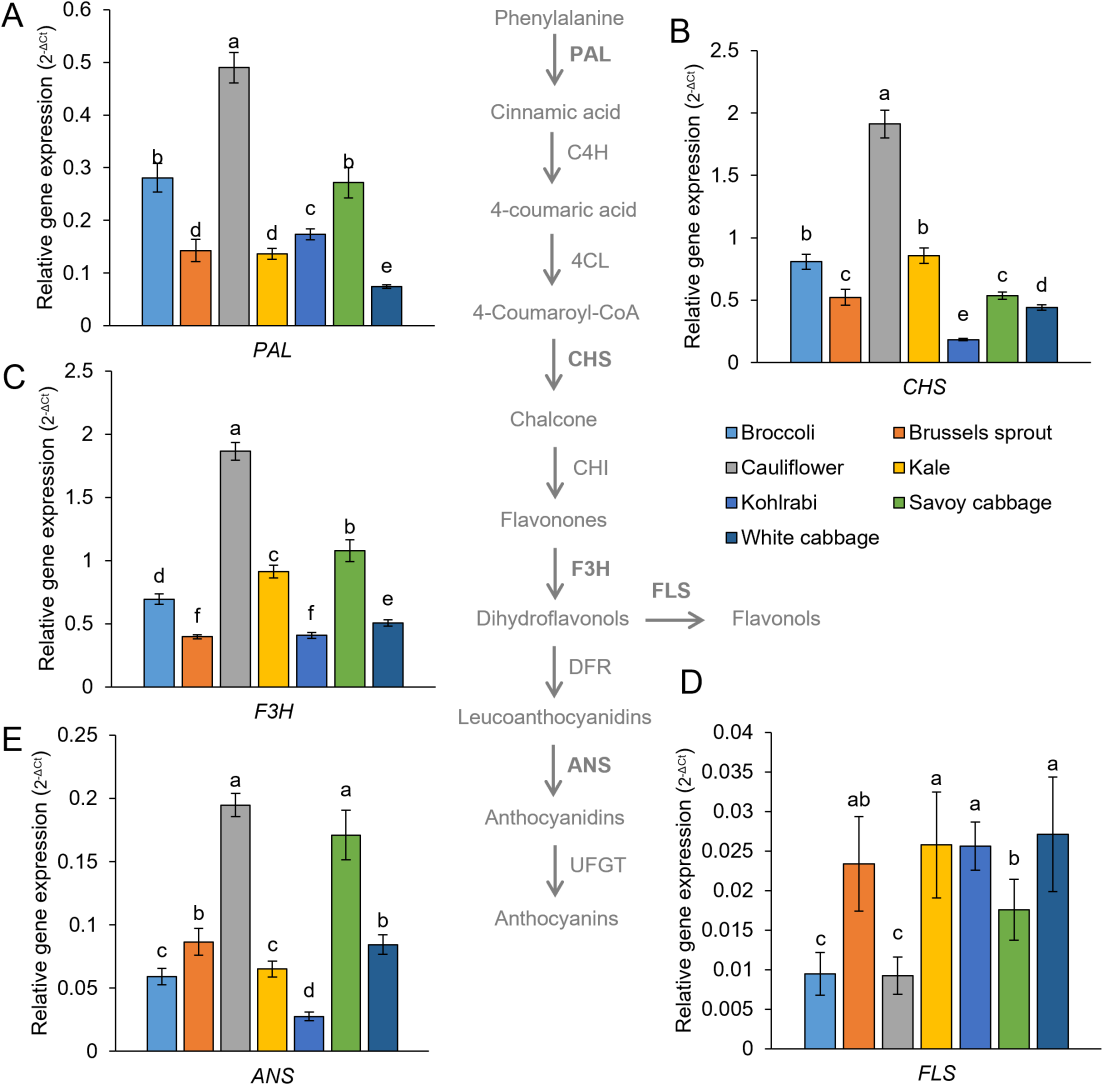
Expression of genes coding for the enzymes of the phenylpropanoid pathway: **(A)** PAL, **(B)** CHS, **(C)** F3H, **(D)** FLS, and **(E)** ANS in seven *Brassica oleracea* varieties, with a representation of the phenylpropanoid pathway. The biosynthesis pathway was adapted from a graphical representation provided in a prior study (Zhu et al., 2015). Values are reported as 2^-ΔCt^, normalised to the expression of two reference genes. Values represent the mean ± standard deviation (*n* = 3). Different letters indicate a significant difference between each variety (ANOVA, Tukey’s test, *p* ≤ 0.05).

Compared to metabolic data for *B. oleracea*, there is only scarce data on the expression of phenolic biosynthesis genes of different *B. oleracea* varieties. Particularly, results combining metabolic and gene expression results are even scarcer. To analyze the regulation of phenolic biosynthesis of *B. oleracea* varieties, we examined the gene expression of several key genes from the phenylpropanoid pathway (**Figure 7**). Interestingly, gene expression of phenylalanine ammonia-lyase (*PAL*), chalcone synthase (*CHS*), and flavanone 3-hydroxylase (*F3H*) correlated with TP, THCA, and TFl levels. This suggests that in *B. oleracea*, the phenylpropanoid pathway is subject to tight transcriptional regulation, indicating the presence of a metabolite-gene regulatory axis. Cauliflower again emerged with the highest expression of *PAL*, *CHS*, *F3H*, as well as anthocyanin synthase (*ANS*). Furthermore, *PAL*, *CHS*, and *F3H* expression levels were found to be mutually correlated. This is to be expected, considering these genes are regulated by mutual transcription factors in the Brassicaceae model *A. thaliana*, namely MYB-A family transcription factors (TFs) (*PAL*), and MYB-B family TFs (*CHS*, *F3H*). Flavonol synthase (*FLS*) is also regulated by MYB-B TFs (Jiang et al., 2023). However, in our study, *FLS* expression levels displayed a negative correlation with THCA, TF, TFl levels, and *PAL* expression. This suggests potential negative feedback caused by high flavonol levels on *FLS* expression or the possibility of additional regulation of *FLS* expression. For example, in grapevine, *FLS* is regulated by the bZIP TF family alongside the MYB-B family. Furthermore, new results for *B. oleracea* are emerging on the influence of microRNAs on the expression of phenylpropanoid pathway genes (Karamat et al., 2024). Those could potentially explain different expression patterns between the phenylpropanoid pathway genes in more detail.

### 3.5 Glucosinolates

**Figure 8:**
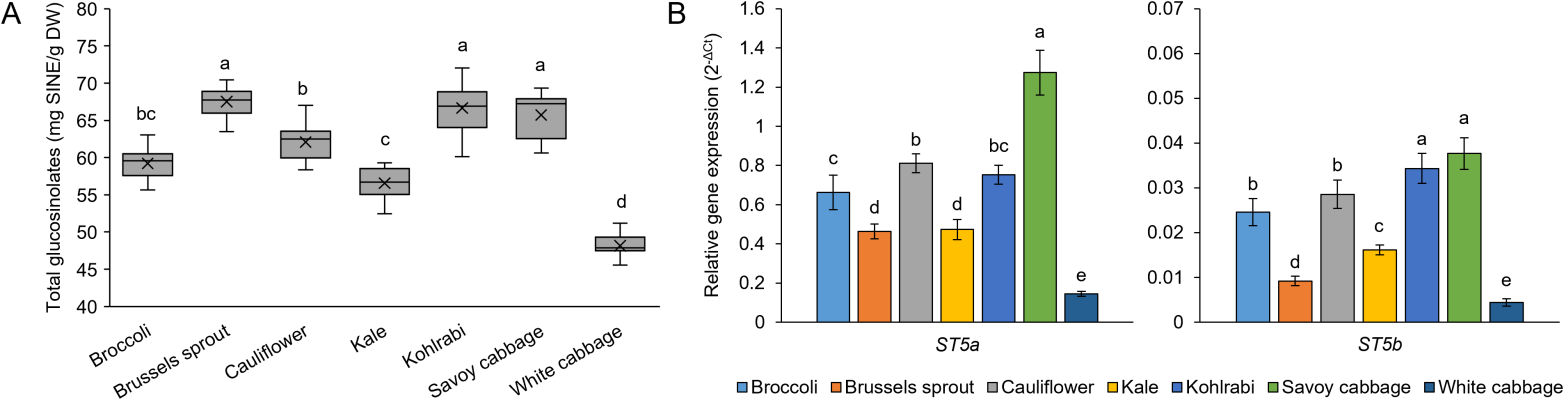
**(A)** Content of total glucosinolates in seven *Brassica oleracea* varieties and **(B)** expression of two sulfotransferase genes (*ST5a* and *ST5b*) involved in glucosinolate biosynthesis. Gene expression values are reported as 2^-ΔCt^, normalised to the expression of two reference genes. Values represent the mean ± standard deviation of nine **(A)** and three **(B)** data points. Different letters indicate a significant difference between each variety (ANOVA, Tukey’s test, *p* ≤ 0.05). SINE, sinigrin equivalents; DW, dry weight.

Another major class of specialized metabolites in Brassicaceae are the glucosinolates, chemically classified as β-thioglucoside-N-hydroxysulfates. Recent data confirm that they also play an important role in stress response (Bansal et al., 2024). Therefore, we analyzed the content of total glucosinolates (TGls) as a putative biomarker of stress tolerance. Previously, broccoli has been shown to decrease TGls under drought (Khan et al., 2011) and salt stress (López-Berenguer et al., 2008; Zaghdoud et al., 2012), but increase TGls under waterlogging conditions (Khan et al., 2012). In white cabbage, heavy metal stress (Kusznierewicz et al., 2012) and biotic stress (*Plasmodiophora brassicae* infection) (Kayum et al., 2021) increased TGls levels. In kale, heavy metals (Jakovljević et al., 2013) and drought stress (Podda et al., 2019) increased TGls, while pest attacks decreased TGls levels (Velasco et al., 2007). In broccoli, drought increased TGls, while flood decreased their level (Šola et al., 2025a). This reveals both stress- and variety-specific responses of TGls levels. Similarly, our study revealed variety-specific differences in TGls levels. Brussels sprout, kohlrabi, and Savoy cabbage had the highest levels, while the lowest level of TGls was recorded in white cabbage (**Figure 8a**). From the nutritional perspective, to maximize the intake of glucosinolates from “baby greens” and their anti-cancer properties and other health benefits, we propose consuming the three varieties with the highest TGls (Wang et al., 2023). We compared our results with the previous comparative studies. As in the present study, in our previous work, Savoy cabbage and Brussels sprout had the highest TGls, followed by cauliflower (Šola et al., 2024b). Another study also recorded the highest TGls in Brussels sprout, followed by cauliflower, kale, and broccoli, similar to our results (Kushad et al., 1999). The lowest values among the tested varieties were also recorded in white cabbage (Castro et al., 2004), which is consistent with our analysis. Interestingly, TGls levels correlated with photosynthetic pigment values, which has also been previously documented in cabbage and baby mustard (Sun et al., 2018; Pan et al., 2023). Their content could be co-regulated by plant hormones, considering both pathways are known to be influenced by hormone-related signaling. Salicylic acid, jasmonic acid, ethylene, and other hormones can regulate both the glucosinolate biosynthesis (Yan and Chen, 2007) and the biosynthesis of photosynthetic pigments (Müller and Munné-Bosch, 2021).

Glucosinolate biosynthesis in *B. oleracea* occurs by two separate pathways, i.e., from methionine and tryptophan. Glucosinolates from the methionine pathway are known as aliphatic, while those from tryptophan are indolic glucosinolates (Sánchez-Pujante et al., 2019). To investigate the glucosinolate pathway on the transcriptional level, we analyzed the gene expression of two sulfotransferase (*ST5a* from the tryptophan pathway and *ST5b* from the methionine pathway) genes from the glucosinolate biosynthetic pathways (**Figure 8b**). We selected these genes because they catalyze the last steps of their respective pathways before the branching to individual glucosinolates, and are also known to be influenced at the transcriptional level in stress conditions (Sánchez-Pujante et al., 2019). Interestingly, relative expression levels for *ST5a* were higher than expression levels for *ST5b*. The reason behind this could be that in *B. oleracea*, alongside ST5b, there is another sulfotransferase (ST5c) in the methionine pathway. Also, we recorded a mutual correlation between *ST5a* and *ST5b*, despite them being regulated by different TFs (MYB51, MYB122, MYB34 for *ST5a* and MYB28, MYB29, MYB76 for *ST5b*) (Bansal et al., 2024), pointing to a probable higher level of master regulation. We recorded strong, although not statistically significant, correlations between TGls levels and gene expression. Despite that, it can be observed that in white cabbage and kale, which had the lowest TGls levels, the lowest expression levels of *ST5a* and *ST5b* were recorded. Also, the highest gene expression was recorded in Savoy cabbage for *ST5a*, and Savoy cabbage and kohlrabi for *ST5b*. However, interestingly, gene expression of both *ST5a* and *ST5b* was low in Brussels sprout, despite a high TGls level, which was probably why a correlation between the three was not established. Still, as corroborated by previous results (Sotelo et al., 2016), we propose these genes as candidate biomarkers of the glucosinolate pathways for further analysis, coupled with investigations of individual glucosinolates. Such compound-specific analyses, utilizing techniques such as HPLC-DAD or LC-MS/MS, could offer valuable insights into the regulation of biosynthesis of individual glucosinolates and specific differences across glucosinolate subclasses.

### 3.6 Chemometric analysis

**Figure 9:**
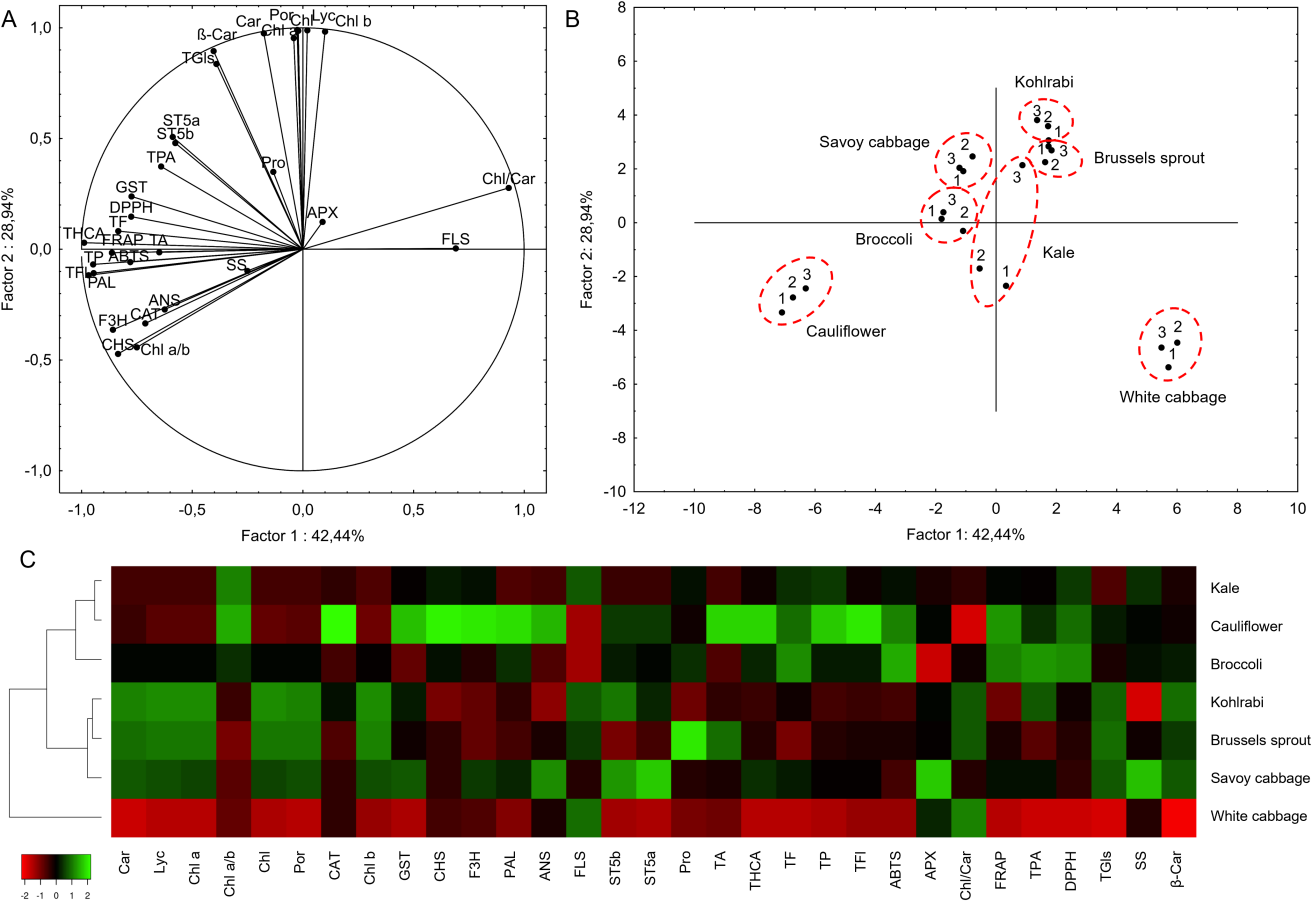
Principal component analysis (PCA) based on all parameters tested: **(A)** loading plot of the measured parameters and **(B)** score plot separating the groups based on the measured parameters. Two factors (factors 1 and 2), which explained the highest portions of the observed variance, were plotted against each other. **(C)** Heatmap with applied hierarchical clustering based on Euclidean distance. A heatmap was prepared using the Heatmapper server (Babicki et al., 2016). ABTS, 2, 2`-azino-bis(3-ethylbenzothiazoline-6-sulfonic acid) radical scavenging assay; *APX*, ascorbate peroxidase; *ANS*, anthocyanin synthase; β-Car, *β-*carotene; Car, total carotenoids; *CAT*, catalase; Chl, total chlorophylls; Chl a, chlorophyll *a*; Chl b, chlorophyll *b*; *CHS*, chalcone synthase; DPPH, 2; 2-diphenyl-1-picrylhydrazyl radical scavenging assay; *F3H*, flavanone 3-hydroxylase; FRAP, ferric ion reducing antioxidant power assay; *FLS*, flavonol synthase; *GST*, glutathione *S*-transferase; Lyc, lycopene; SS, soluble sugars; *ST*, sulfotransferase; *PAL*, phenylalanine ammonia-lyase; Pro, proline; Por, total porphyrins; TA, total anthocyanins; TF, total flavonoids; TFl, total flavonols; TGls, total glucosinolates; THCA, total hydroxycinnamic acids; TP, total phenolics; TPA, total phenolic acids.

Finally, we used PCA and HCA to perform the chemometric analysis that summarizes the physiological parameter data obtained for the varieties in our study. The results of the PCA were visualized by plotting factors 1 and 2, which explained 42.44% and 28.94% of the variance, respectively (**Figure 9**). The auxiliary results of the PCA analysis are provided in **Supplementary Table S8**. The biological replicates of each variety analyzed were grouped together in most cases, indicating low inter-experiment variance, except for the third kale replicate, which separated from the other two based on factor 2. The results of the PCA led to the conclusion that phenolic as well as antioxidative parameters (primarily THCA, TFl, TP, PAL, FRAP, TF, F3H, DPPH, CHS, GST, ABTS), followed by photosynthesis- and glucosinolates-related parameters (Chl, Lyc, Por, Chl b, Chl a, Car, β-Car, TGls, ST5a, ST5b), have the highest influence on the separation of the varieties. Based on the first factor, cauliflower on one end, and white cabbage on the other, separated the most from all other varieties, which was due to the phenolic levels, phenolic-related gene expression, and antioxidative parameters, of which cauliflower had the highest levels, while white cabbage had the lowest (**Supplementary Table S8b**). Based on the second factor, the factors that contributed the most to the separation were the photosynthetic and glucosinolate-related parameters. Kohlrabi separated the most on one end with Brussels sprout, Savoy cabbage with broccoli and kale in the middle, and cauliflower and white cabbage on the other end, aligning with the levels of those parameters reported in the varieties tested.

The results of the HCA aligned well with the results of the PCA (**Figure 9c**). Due to the lowest reported values of most parameters recorded, white cabbage formed a separate cluster from all other varieties. The rest of the varieties were separated into two clusters. One cluster was comprised of kohlrabi, Brussels sprout, and Savoy cabbage, with Savoy cabbage, similar to the PCA, further separating from the two. This cluster was primarily formed based on the highest levels of photosynthetic pigments. The second cluster was comprised of kale, cauliflower, and broccoli, with broccoli further separating from the other two. This cluster was primarily formed based on the highest values of phenolic-related parameters, while the separation of broccoli was likely due to the lowest *APX* and *FLS* gene expression. Since white cabbage had the lowest levels of most of the parameters tested among all varieties, this could point to its lower stress tolerance. Despite these low values, white cabbage still possesses the machinery to deal with stress conditions. This does not exclude white cabbage from having high concentrations of other important stress tolerance factors that were not examined in the present study, such as heat-shock proteins (Al-Whaibi, 2011), or other classes of less-represented stress-responsive molecules. At the other end of the spectrum, cauliflower emerged as the one variety with the highest levels of phenolic parameters, setting it apart from the other varieties. This could indicate that cauliflower, if challenged with stresses that induce severe oxidative stress conditions, could be the most resistant to such conditions due to the highest endogenous antioxidant concentrations. Photosynthetic- and glucosinolate-related parameters separated white cabbage from the other varieties and positioned kohlrabi, Brussels sprout, and Savoy cabbage as the varieties with the highest levels, which could point to the highest tolerance to stresses that severely impact photosynthetic processes, and stresses where glucosinolates play an important role in stress tolerance.

## 4 Conclusion

In this work, parameters that have been reported as being important for stress responses in plants have been determined, but without the application of actual stressors. Potential mechanisms involved in the plant adjustments to stress can include regulation of transcripts and metabolites, but also signaling molecules and growth regulators such as plant hormones. The latter are important features since plants always need to balance growth and defense mechanisms under stress responses (Zhang et al., 2020). Energy and resource constraints typically account for an inevitable trade-off between growth and stress resistance. Therefore, plants under stress must redirect their energy and resources from growth to a stress response.

We observed a high variability between the varieties and the parameters tested. Therefore, our results set the ground for future studies to deal with the observed variability accordingly. Based on our study, future research could be directed to analyzing the intra-varietal differences between genotypes or accession belonging to a single variety. Mechanisms associated with plasticity under stress conditions are an important feature that needs to be taken into account if the observations from our study should be possibly implemented. Phenotypic plasticity can be defined as the ability of an organism to adjust a specific phenotype in response to changes in its environment (Alseekh et al., 2025). The understanding of phenotypic plasticity will be crucial for predicting changes in, e.g., crop productivity under adverse environmental conditions (Gratani, 2014).

We concluded that not just a single basal parameter or set of parameters can be used as an indicator of stress tolerance. Therefore, in future study design, when assessing the stress tolerance to specific types of stresses, we propose using an array of parameters to test, as was the one utilized in our study. The next step, on the basis of the present study, will be to perform different stress treatments on the tested *B. oleracea* varieties to experimentally validate the indications of stress tolerance illustrated in this study to gain deeper insights into the regulation of stress response in *B. oleracea*. From the patterns observed here, we believe there will also be variety-specific and, additionally, stress-specific responses of different parameters to specific stresses applied. The results on the impact of waterlogging stress on the same selection of varieties were recently published. The study employed younger plants (30 days old) compared to the present study, with trends slightly differing, thus also confirming a substantial influence of the developmental stage on the basal levels of stress parameters, alongside experimentally corroborating our hypothesis on the variety-dependent impact of specific stress conditions (Davosir et al., 2025). However, a definition of adaptation or natural selection that occurs in the environment in populations may not be possibly usable for a selection of different varieties that have, e.g., been bred for specific consumer quality as used in our study. Other work has, for example, investigated the plasticity of the responses to drought stress by comparing crop vs. wild relative species (Lupo and Moshelion, 2024).

Another major outcome of our present research was the establishment of a link between metabolic data and gene expression values of corresponding biosynthesis genes for phenolics and glucosinolates. Future research should confirm whether the observed variability of gene expression patterns between the varieties could be traced to the genetic or epigenetic level. Further studies should also include the analyses of individual phenolic compounds and glucosinolates, as this would allow a more precise understanding of their biosynthetic regulation, metabolic interactions, and potential contributions to the observed varietal differences. Since all varieties of *B. oleracea* originate from different wild cabbage tissues (Swarup et al., 2021), we will aim to investigate if the differences and variety-specific gene expression in our selected varieties could be traced to the gene expression of corresponding origin tissues in wild cabbage, or perhaps this link is lost through the hundreds of years of domestication and breeding of this important and diverse plant species.

## Data Availability

The original contributions presented in the study are included in the article/**Supplementary Material**. Further inquiries can be directed to the corresponding author.
