# Supplementary material for: Variances in physiological parameters associated with stress tolerance between seven Brassica oleracea varieties
Source: Front Plant Sci. 2025 Dec 19;16:1713155. doi: 10.3389/fpls.2025.1713155 (PMC12757237; doi:10.3389/fpls.2025.1713155)
Supplement: Supplementary file 1 [file DataSheet1.docx]

Supplementary Material

# Supplementary Methods

## Estimation of proline content

Proline content was evaluated with a method introduced by Bates et al. (1973), which was later adapted to avoid using toluene (Carillo and Gibon, 2011). Volumes used in the method were adapted for *Brassica* samples (Ljubej et al., 2021), and the method was further optimized (Davosir et al., 2024b). The supernatant (60 μL) was mixed with 600 μL of ninhydrin reagent (1% ninhydrin, 60% acetic acid, 20% ethanol) and the mixture was homogenized on a vortex mixer and incubated for 20 min at 95 °C in a Thermomixer (Eppendorf, Hamburg, Germany). The modification compared to (Ljubej et al., 2021) included that every sample had a separate blank to minimize the absorbance of other compounds present in the extract. For each blank, the same volume of the extract was mixed with 600 μL of 60% acetic acid and 20% ethanol, and the reaction conditions were the same as described above. After the incubation, 200 μL of the sample was transferred in three technical replicates on a 96-well plate, and the colour intensity was quantified by measuring the absorbance at 520 nm on an Infinite^®^ 200 PRO microplate reader (Tecan, Männedorf, Switzerland). The measured absorbance of the blank was subtracted from the absorbance of the samples, and the proline content in the samples was calculated indirectly based on the calibration curve of standard *L*-proline solutions of known concentrations (1.25-0.009 mg/mL). The results were expressed as mg of *L*-proline per g of DW.

## Estimation of soluble sugars content

Soluble sugar content in ethanolic extracts was evaluated using the method from Dubois et al. (1956), adapted for smaller volumes (Davosir et al., 2024b). The volume of 200 μL of each extract diluted to 1 mg/mL was mixed with 100 μL of 5% phenol (v/v) and 500 μL of concentrated H_2_SO_4_. Then, samples were homogenized on a vortex mixer and incubated for 10 min at RT, after which the samples were incubated for 10 min in a water bath at 30 °C. After the incubation, 200 μL of the sample was transferred in three technical replicates on a 96-well plate and the colour intensity was quantified by measuring the absorbance at 490 nm on an Infinite^®^ 200 PRO microplate reader (Tecan, Männedorf, Switzerland). As blank, 70% ethanol was used instead of the extract. The measured absorbance of the blank was subtracted from the absorbance of the samples, and the soluble sugar content in the samples was calculated indirectly as mg of sucrose equivalents (SE) per g of DW based on the calibration curve of standard sucrose solutions of known concentrations (5-0.01 mg/mL).

## Estimation of total phenolic content

For the determination of total phenolic content, the method according to Singleton and Rossi (1965) was used, which was modified further for smaller volumes as reported previously (Vujčić Bok et al., 2023). Extracts (10 μL) were mixed with 790 μL of deionized water and 50 μL of Folin-Ciocaulteu (FC) reagent, and the mixture was homogenized on a vortex mixer. Then, 150 μL of 1.88 M Na_2_CO_3_ was added, and the mixture was homogenized and incubated for 30 min at 45 °C in an incubator. After the incubation, 200 μL of the sample was transferred in three technical replicates on a 96-well plate and the colour intensity was quantified by measuring the absorbance at 765 nm on an Infinite^®^ 200 PRO microplate reader (Tecan, Männedorf, Switzerland). As blank, 70% ethanol was used instead of the extract. The measured absorbance of the blank was subtracted from the absorbance of the samples, and the total phenolics content in the samples was calculated indirectly as mg of gallic acid equivalents (GAE) per g of DW based on the calibration curve of standard gallic acid solutions of known concentrations (5-0.05 mg/mL).

## Estimation of total phenolic acid content

Phenolic acid content was estimated using the method described in (Jain et al., 2017), adapted for smaller volumes and with an appropriated sample dilution (Davosir et al., 2024a). Extract (70 μL) was mixed with 140 μL of 0.5 M HCl and 140 μL of Arnow reagent (10% NaNO_2_, 10% Na_2_MoO_2_ in H_2_O), 140 μL of 8.5% NaOH and 210 μL of H_2_O. After homogenizing the sample on a vortex mixer, 200 μL of each sample was transferred in three technical replicates on a 96-well plate, and the colour intensity was quantified by measuring the absorbance at 490 nm on an Infinite^®^ 200 PRO microplate reader (Tecan, Männedorf, Switzerland). As blank, 70% ethanol was used instead of the extract. The measured absorbance of the blank was subtracted from the absorbance of the samples, and the phenolic acid content in the samples was calculated indirectly as mg of caffeic acid equivalents (CAE) per g of DW based on the calibration curve of standard caffeic acid solutions of known concentrations (2-0.1 mg/mL).

## Estimation of total flavonoid content

For the determination of total flavonoid content, the method introduced by Zhishen et al. (1999) was used, which was adapted for smaller volumes as described (Davosir et al., 2024a), with an appropriated sample dilution. Extracts (70 μL) were mixed with 280 μL of deionized water and 21 μL of 5% NaNO_2_ solution and the mixture was homogenized on a vortex mixer and incubated for 5 min at room temperature (RT). Then, 21 μL of 10% AlCl_3_ was added, and the mixture was homogenized and incubated for 6 min at RT. After incubation, 140 μL of 1 M NaOH and 168 μL of deionized water were added. Afterwards, 200 μL of each sample was transferred in three technical replicates on a 96-well plate and the colour intensity was quantified by measuring the absorbance at 510 nm on an Infinite^®^ 200 PRO microplate reader (Tecan, Männedorf, Switzerland). As blank, 70% ethanol was used instead of the extract. The measured absorbance of the blank was subtracted from the absorbance of the samples, and the total flavonoid content in the samples was calculated indirectly as mg of quercetin equivalents (QE) per g of DW, based on the calibration curve of standard quercetin solutions of known concentrations (1-0.0125 mg/mL).

## Estimation of total anthocyanin content

The content of total anthocyanins in the extracts was evaluated using the method modified from Tušek et al. (2016), with slight modifications. An appropriate sample dilution of *Brassica* samples was obtained by mixing the volume of 150 μL of each extract with 450 μL of 70% (v/v) ethanol and 50.4 μL of concentrated HCl. The samples were briefly mixed on a vortex mixer and incubated for 60 min in a Thermomixer (Eppendorf, Hamburg, Germany) at 80 °C and 300 rpm. After the incubation, 200 μL of each sample was transferred in three technical replicates on a 96-well plate and the colour intensity was quantified by measuring the absorbance at 520 nm on an Infinite^®^ 200 PRO microplate reader (Tecan, Männedorf, Switzerland). As blank, 70% ethanol was used instead of the extract. The measured absorbance of the blank was subtracted from the absorbance of the samples, and the total anthocyanin content in the samples was calculated indirectly as pelargonidin chloride equivalents (PCE) based on the on the calibration curve of standard solutions of known concentrations (0.3125-0.009765625 mg/mL).

## Estimation of total flavonol and hydroxycinnamic acid content

Total flavonols and hydroxycinnamic acids content was estimated according to the method described in Howard et al. (2003), adapted for smaller volumes with an appropriated sample dilution. Extract (10 μL) was mixed with 40 μL of 70% (v/v) ethanol, 50 μL of HCl (1 g/L in 96% ethanol) and 910 μL of HCl (2 g/L in H_2_O). After homogenizing the sample on a vortex mixer, 200 μL of each sample was transferred in three technical replicates on a 96-well plate and the colour intensity was quantified by measuring the absorbance at 320 nm for hydroxycinnamic acids and 360 nm for flavonols on an Infinite^®^ 200 PRO microplate reader (Tecan, Männedorf, Switzerland). As blank, 70% ethanol was used instead of the extract. The measured absorbance of the blank was subtracted from the absorbance of the samples, and the flavonol content in the samples was calculated indirectly as mg of quercetin equivalents (QE) per g of DW based on the calibration curve of standard quercetin solutions of known concentrations (1-0.00625 mg/mL). The hydroxycinnamic acid content in the samples was calculated indirectly as mg of caffeic acid equivalents (CAE) per g of DW based on the calibration curve of standard caffeic acid solutions of known concentrations (2-0.05 mg/mL).

## Estimation of total glucosinolate content

Total glucosinolate content was evaluated as described in a simplified protocol by Mawlong et al. (2017), which was adapted for smaller volumes. A volume of 50 μL of the extract was mixed with 1 mL 2 mM sodium tetrachloropalladate (58.8 mg sodium tetrachloropalladate + 170 µl concentrated HCl +100 ml deionized water), followed by homogenizing the mixture and incubation at room temperature for 1 h. Afterwards, 200 μL of each sample was transferred in three technical replicates on a 96-well plate and the colour intensity was quantified by measuring the absorbance at 425 nm on an Infinite^®^ 200 PRO microplate reader (Tecan, Männedorf, Switzerland). As blank, 70% ethanol was used instead of the extract. The measured absorbance of the blank was subtracted from the absorbance of the samples, and the total glucosinolate content in the samples was calculated indirectly as mg of sinigrin equivalents (SE) per g of DW, based on the calibration curve of standard sinigrin solutions of known concentrations (1-0.0125 mg/mL).

## Estimation of antioxidative capacity by ABTS assay

Antioxidative capacity of plant extracts was evaluated by the ABTS (2,2'-azino-bis(3-ethylbenzothiazoline-6-sulfonic acid)) assay as described in Re et al. (1999). Prior to analysis, the ABTS^+^ radical solution (88 μL of 140 mM K_2_S_2_O_8_ filled to 5 mL with 7 mM ABTS) was prepared, with absorbance of the solution at 734 nm fixed to 0.7 by diluting it with 96% ethanol (v/v). The absorbance of the solution was monitored using the Nanodrop 2000c spectrophotometer (Thermo Fisher Scientific, Waltham, USA). A smaller extract and standard amounts were used compared to the original study. In a tube, 1 μL of the extract was mixed with 700 μL of ABTS^+^ solution, and the mixture was homogenized on a vortex mixer, and the samples were incubated in the dark for 6 min at RT. After the incubation, 200 μL of each sample were transferred in three technical replicates on a 96-well plate and the colour intensity was quantified by measuring the absorbance at 734 nm on an Infinite^®^ 200 PRO microplate reader (Tecan, Männedorf, Switzerland). As blank, 70% ethanol was used instead of the extract. Based on the recorded absorbance values, the inhibition of the ABTS^+^ radical (%) was calculated using the equation:

$$\% inhibition=\frac{\left( A_{blank}-A_{sample} \right)\times100}{A_{blank}}$$

where: A_blank_ – absorbance of the blank, A_sample_ – absorbance of the sample.

The results were presented indirectly through the equivalents of antioxidative capacity of the standard antioxidant *L*-ascorbic acid, based on the calibration curve of standard solutions of known concentrations (5-0.005 mg/mL), and the value was expressed as mg *L*-ascorbic acid equivalents (AAE)/g DW.

## Estimation of antioxidative capacity by DPPH assay

Determination of antioxidative capacity by DPPH (2,2-diphenyl-1-picrylhydrazyl) assay was carried out according to Germanò et al. (2002), with modified sample and standard volumes used in the assay. The volume of 3.5 μL of each sample was mixed with 700 μL of DPPH solution (0.1 mM, in 96% ethanol), and the solution was homogenized on a vortex mixer and incubated in the dark for 30 min at RT. After the incubation, 200 μL of each sample were transferred in three technical replicates on a 96-well plate and the colour intensity was quantified by measuring the absorbance at 520 nm on an Infinite^®^ 200 PRO microplate reader (Tecan, Männedorf, Switzerland). As blank, 70% ethanol was used instead of the extract. Based on the recorded absorbance values, the inhibition of the DPPH radical (%) was calculated using the equation:

$$\% inhibition=\frac{\left( A_{blank}-A_{sample} \right)\times100}{A_{blank}}$$

where: A_blank_ – absorbance of the blank, A_sample_ – absorbance of the sample.

The results were presented indirectly through the equivalents of antioxidative capacity of the standard antioxidant *L-*ascorbic acid, based on the calibration curve of standard solutions of known concentrations (5-0.005 mg/mL), and the value was expressed as mg *L*-ascorbic acid equivalents (AAE)/g DW.

## Estimation of antioxidative capacity by FRAP assay

Determination of antioxidative capacity by FRAP (ferric reducing/antioxidant power) assay was carried out according to Benzie and Strain (1999). The FRAP reagent was prepared by mixing the 300 mM acetate buffer, pH 3.6, 10 mM TPTZ solution in 40 mM HCl and 20 mM FeCl_3_ in the ratio 10:1:1. An optimized final volume of 1 μL of each sample was mixed with 650 μL of FRAP reagent and the solution was homogenized on a vortex mixer and incubated for 4 min at RT. After the incubation, 200 μL of each sample were transferred in three technical replicates on a 96-well plate and the colour intensity was quantified by measuring the absorbance at 593 nm on an Infinite^®^ 200 PRO microplate reader (Tecan, Männedorf, Switzerland). As blank, 70% ethanol was used instead of the extract. Based on the recorded absorbance values, the reduction percentage (%) was calculated using the equation:

$$\% reduction=\frac{\left( A_{sample}-A_{blank} \right)\times100}{A_{sample}}$$

where: A_blank_ – absorbance of the blank, A_sample_ – absorbance of the sample.

The results were presented indirectly through the equivalents of antioxidative capacity of the standard antioxidant *L-*ascorbic acid, based on the calibration curve of standard solutions of known concentrations (5-0.005 mg/mL), and the value was expressed as mg *L*-ascorbic acid equivalents (AAE)/g DW.

## References

Bates, L. S., Waldren, R. P., and Teare, I. D. (1973). Rapid determination of free proline for water-stress studies. *Plant Soil* 39, 205–207. doi: 10.1007/BF00018060

Benzie, I. F. F., and Strain, J. J. (1999). “Ferric reducing/antioxidant power assay: Direct measure of total antioxidant activity of biological fluids and modified version for simultaneous measurement of total antioxidant power and ascorbic acid concentration,” in *Methods in Enzymology*, (Elsevier), 15–27. doi: 10.1016/S0076-6879(99)99005-5

Carillo, P., and Gibon, Y. (2011). Extraction and determination of proline. Available at: https://www.researchgate.net/publication/211353600_PROTOCOL_Extraction_and_determination_of_proline (Accessed November 5, 2025).

Davosir, D., Šola, I., Ludwig-Müller, J., and Šeruga Musić, M. (2024a). *Flavescence dorée* strain-specific impact on phenolic metabolism dynamics in grapevine (*Vitis vinifera*) throughout the development of phytoplasma infection. *J. Agric. Food Chem.* 72, 189–199. doi: 10.1021/acs.jafc.3c06501

Davosir, D., Šola, I., and Šeruga Musić, M. (2024b). Physiological responses of grapevine (*Vitis vinifera* var. ‘Pinot gris’) affected by different *flavescence dorée* genotypes: dynamics through the development of phytoplasma infection. *J. Plant Dis. Prot.* 131, 1411–1425. doi: 10.1007/s41348-024-00937-7

Dubois, M., Gilles, K. A., Hamilton, J. K., Rebers, P. A., and Smith, F. (1956). Colorimetric method for determination of sugars and related substances. *Analytical Chemistry* 28, 350–356. doi: 10.1021/ac60111a017

Germanò, M. P., De Pasquale, R., D’Angelo, V., Catania, S., Silvari, V., and Costa, C. (2002). Evaluation of extracts and isolated fraction from *Capparis spinosa* L. buds as an antioxidant source. *J. Agric. Food Chem.* 50, 1168–1171. doi: 10.1021/jf010678d

Howard, L. R., Clark, J. R., and Brownmiller, C. (2003). Antioxidant capacity and phenolic content in blueberries as affected by genotype and growing season. *Journal of the Science of Food and Agriculture* 83, 1238–1247. doi: 10.1002/jsfa.1532

Jain, R., Rao, B., and Tare, A. B. (2017). Comparative analysis of the spectrophotometry based total phenolic acid estimation methods. *J Anal Chem* 72, 972–976. doi: 10.1134/S106193481709009X

Ljubej, V., Karalija, E., Salopek-Sondi, B., and Šamec, D. (2021). Effects of short-term exposure to low temperatures on proline, pigments, and phytochemicals level in kale (*Brassica oleracea* var. *acephala*). *Horticulturae* 7, 341. doi: 10.3390/HORTICULTURAE7100341

Mawlong, I., Sujith Kumar, M. S., Gurung, B., Singh, K. H., and Singh, D. (2017). A simple spectrophotometric method for estimating total glucosinolates in mustard de-oiled cake. *International Journal of Food Properties* 20, 3274–3281. doi: 10.1080/10942912.2017.1286353

Re, R., Pellegrini, N., Proteggente, A., Pannala, A., Yang, M., and Rice-Evans, C. (1999). Antioxidant activity applying an improved ABTS radical cation decolorization assay. *Free Radical Biology and Medicine* 26, 1231–1237. doi: 10.1016/S0891-5849(98)00315-3

Singleton, V. L., and Rossi, J. A. (1965). Colorimetry of total phenolics with phosphomolybdic-phosphotungstic acid reagents. *Am J Enol Vitic.* 16, 144–158. doi: 10.5344/ajev.1965.16.3.144

Tušek, M., Curman, M., Babić, M., and Tkalec, M. (2016). Photochemical efficiency, content of photosynthetic pigments and phenolic compounds in different pitcher parts of sarracenia hybrids. *Acta Botanica Croatica* 75, 179–185. doi: 10.1515/botcro-2016-0036

Vujčić Bok, V., Gerić, M., Gajski, G., Gagić, S., and Domijan, A.-M. (2023). Phytotoxicity of bisphenol A to *Allium cepa* root cells is mediated through growth hormone gibberellic acid and reactive oxygen species. *Molecules* 28, 2046. doi: 10.3390/molecules28052046

Zhishen, J., Mengcheng, T., and Jianming, W. (1999). The determination of flavonoid contents in mulberry and their scavenging effects on superoxide radicals. *Food Chemistry* 64, 555–559. doi: 10.1016/S0308-8146(98)00102-2
